# Effect of Oxaliplatin-Loaded Poly (d,l-Lactide-*co*-Glycolic Acid) (PLGA) Nanoparticles Combined with Retinoic Acid and Cholesterol on Apoptosis, Drug Resistance, and Metastasis Factors of Colorectal Cancer

**DOI:** 10.3390/pharmaceutics12020193

**Published:** 2020-02-23

**Authors:** Ana Luiza C. de S. L. Oliveira, Raimundo Fernandes de Araújo Júnior, Thaís Gomes de Carvalho, Alan B. Chan, Timo Schomann, Filippo Tamburini, Lioe-Fee de Geus-Oei, Luis J. Cruz

**Affiliations:** 1 Postgraduate Program in Health Science, Federal University of Rio Grande do Norte (UFRN), 59078 970 Natal, RN, Brazil; analuiza_oliveira@outlook.com (A.L.C.d.S.L.O.); thaisbida2011@hotmail.com (T.G.d.C.); 2 Translational Nanobiomaterials and Imaging, Department of Radiology, Leiden University Medical Center, Albinusdreef 2, 2333 ZA Leiden, The Netherlands; t.schomann@lumc.nl; 3 Postgraduate Program in Functional and Structural Biology, Department of Morphology, Federal University of Rio Grande do Norte (UFRN), 59078 970 Natal, RN, Brazil; 4 Percuros B.V, Zernikedreef 8, 2333 CL Leiden, The Netherlands; alanchan@clara.net (A.B.C.); tambu.91@hotmail.it (F.T.); 5 Nuclear Medicine, Department of Radiology, Leiden University Medical Center, 2333 ZA Leiden, The Netherlands; l.f.de_geus-oei@lumc.nl

**Keywords:** PLGA nanoparticles, oxaliplatin, colorectal cancer, drug resistance, apoptosis

## Abstract

Apoptosis signaling pathways, drug resistance, and metastasis are important targets to develop new cancer treatments. We developed cholesterol-coated Poly(d,l-Lactide-*co*-Glycolic Acid) (PLGA) nanoparticles for effective encapsulation and delivery of retinoic acid and oxaliplatin to analyze their antitumor activity in colorectal cancer. The cell viability and proliferation of tumoral cells lines (CT-26 and SW-480) decreased when compared to control in vitro after treatment with the nanoparticles. In addition, apoptosis of CT-26 cells increased. Importantly, cytoprotection of nontumor cells was detected. Expression of pro-apoptotic proteins was upregulated, while anti-apoptotic proteins were downregulated either in vitro or in vivo. In addition, drug resistance and metastasis factors were downregulated in vivo. Human colorectal tumors that highly expressed BCL-2 and Ki-67 had a greater tendency towards death within 60 months. Our results show that loading oxaliplatin combined with retinoic acid and cholesterol in a nanoparticle formulation enables determination of optimal antitumor activity and subsequent treatment efficacy.

## 1. Introduction

Colorectal cancer (CRC) is the third most commonly occurring malignancy around the world with significant morbidity and mortality rates. Every year, 1.2 million people are diagnosed with CRC [1,2,3,4]. Evasion of apoptosis is one of the hallmarks of cancer, in general, and correlated to drug resistance and metastatic spread, which urges the development of new drugs [5,6,7]. Apoptosis occurs through the extrinsic pathway or intrinsic pathway. Both pathways converge at the activation of caspase-3, which then induces other caspases downstream of caspase-3 and eventually leads to apoptosis of the cancer cells [6,8].

Oxaliplatin (OXA) is used to treat CRC, in combination with other drugs. OXA binds to nucleophilic molecules and forms adducts that inhibit gene transcription [9,10]. Unfortunately, tumor resistance to OXA treatment is not uncommon and can occur via a mutation in the intrinsic apoptosis pathway, which results in metastatic spread [7,11]. In addition, toxic effects are also observed. Neurotoxicity through reversible sensory neuropathy or chronic cumulative neuropathy is frequently observed [12].

To overcome these limitations of OXA, a drug delivery system (DDS) that can increase efficacy and reduce adverse effects by increasing the circulation time and bioavailability of the drug can be used [13]. This would result in a high resistance to clearance and increased concentration of the drug in the target tissues, thus, requiring lower doses of drugs [14]. One of the most widely used DDSs is the biodegradable and biocompatible copolymer poly (d,l-lactic-*co*-glycolic acid) (PLGA) due to its metabolite monomers: Lactic acid and glycolic acid. This polymer is approved by the Food and Drug Administration (FDA) and the European Medicine Agency (EMA) in several clinically applied DDS [15]. Nanoparticles (NPs) are a form of DDS. The commonly utilized techniques for the preparation of NPs are the nanoprecipitation technique [16], the emulsification solvent extraction/evaporation method [17,18], the emulsification solvent diffusion [19,20], and the double emulsion solvent evaporation [21]. The choice of a particular method of encapsulation is mainly determined by the solubility and molecular stability of the drug. In this study, we prepared NPs made from PLGA using the emulsification solvent extraction/evaporation method.

Cholesterol (CHO) is involved in the endocytosis of materials as well as cancer proliferation and metastasis. Different types of cells have different amounts of cholesterol in their membranes. This difference becomes clearer when comparing healthy cells with cancer cells. Cancer cells have a high proliferation rate. Thus, the membranes of these cells are synthesized rapidly, which requires more nutrients and leads to higher CHO content of the membrane [22,23].

Retinoic acid (RA) binds to the heterodimers of the retinoic acid receptor (RARs) and the retinoid X receptor (RXRs) present in the nuclear membrane of cancer cells, which leads to growth inhibition, differentiation, or apoptosis in these cells. Studies have shown that, when used in combination with chemotherapeutic agents such as OXA, those characteristics of RA lead to increased cytotoxicity and decreased side effects via synergistic action of therapeutic agents [24,25,26].

In this study, PLGA, OXA, CHO, and RA were combined to formulate distinct NPs for the delivery of drugs to targeted cells. It is expected that CHO-coated PLGA NPs have an enhanced tumor-targetability when compared to exclusive PLGA NPs. Therefore, the aim of the present work was to evaluate the biological efficacy of OXA against CRC cells when co-encapsulated with RA in CHO-coated PLGA NPs.

## 2. Materials and Methods

### 2.1. Preparation of PLGA Nanoparticles

The PLGA NPs were synthesized using a solvent extraction/evaporation method [27,28]. We hypothesized the emulsification solvent extraction/evaporation technique could result in better encapsulation yield of hydrophilic (OXA) and hydrophobic (RA) molecules simultaneously. Briefly, 100 mg of PLGA (Corbion, Amsterdam, The Netherlands) were dissolved in 3 mL of dichloromethane (DCM). Depending on the PLGA NPs, the following substances were added: 20 mg of OXA (European Pharmacopoeia Reference Standards), 0.5 mg of IR-780 iodide (Sigma-Aldrich, St. Louis, MO, USA), and/or 20 mg of RA (Sigma-Aldrich, St. Louis, MO, USA). The solution containing the NPs constituents was added dropwise to 25 mL of aqueous 2.5% (*w*/*v*) polyvinyl alcohol (PVA) and emulsified using a sonicator (250 watt, Sonifier 250; Branson, Danbury, CT, USA). PVA acted as a surfactant molecule, which stabilized the emulsion nanoparticles, avoided aggregation, and prevented them from coalescing with each other. In addition, PVA acted as an effective stabilization and the PVA surfactant molecules allowed us to achieve small particle size and narrow size distribution. A very low concentrations of PVA remained on the surface of nanoparticles [29]. The solution was transferred to a new vial that contained an air-dried solution of 20 mg CHO (Avati Polar Lipids, Alabaster, AL, USA), dissolved in 0.4 mL of chloroform, and homogenized by sonication. After evaporating the solvent, the NPs were collected by centrifugation and lyophilized for 3 days. The concentration of each encapsulated constituent was determined by reverse phase high-performance liquid chromatography (RP-HPLC), as described elsewhere [30,31].

### 2.2. Physicochemical Properties of PLGA NPs

PLGA NPs were characterized by average size, polydispersity index, and surface charge (zeta-potential) by dynamic light scattering. PLGA NP samples were measured for size using a Zetasizer (Nano ZS, Malvern Ltd., Worcestershire, UK), and were analyzed for surface charge by laser Doppler electrophoresis on the same device.

### 2.3. Atomic Force Microscope (AFM)

The shape and surface of PLGA NPs were visualized by AFM. The nanoparticle dispersion was deposited and left to dry overnight on the mica for analysis using a JPK NanoWizard^®^ 3 NanoOptics AFM System (JPK BioAFM Business, Berlin, Germany) with intermittent contact mode cantilevers (70 kHz). Raw data (height (measured) trace) obtained from the microscope were processed with JPKSPM Data Processing software using the plane flattening algorithm.

### 2.4. Viability Assay

An initial screening was performed by adding free OXA (5 μg/mL, 10 μg/mL, 25 μg/mL, 50 μg/mL, 100 μg/mL, and 200 μg/mL) to the CT-26 plated in a 96-well plate. Then a second assay was performed using free OXA as well as all PLGA NPs formulation systems (Table 1) at concentrations of 5 μg/mL, 10 μg/mL, and 25 μg/mL. Culture medium without drug (negative) and 25% DMSO (positive) were used as controls. After incubation for 24, 48, or 72 h, 20 μL/well of MTS [3-(4,5-dimethylthiazol-2-yl)-5-(3-carboxymethoxyphenyl)-2-(4-sulfophenyl)-2H-tetrazolium, inner salt] (Promega Corporation, Madison, WI, USA) solution was added and incubated for 3 h. Absorbance was measured at 490 nm.

For SW-480 cells, Hoechst labelling and flow cytometric analysis were performed. The cells were cultured in a 96-well plate for 24 h. Next, free OXA (5 μg/mL, 25 μg/mL, 50 μg/mL), NPs 1, and NPs 2 (5 μg/mL, 25 μg/mL, 50 μg/mL) were added and incubated for 24 and 48 h. Afterwards, the cells were labelled with Hoechst, measured with a flow cytometer BD LSR II (BD Biosciences, San Jose, CA, USA) and analyzed with FlowJo (version 10.1, BD Life Sciences, Franklin Lakes, NJ, USA).

### 2.5. Detection of Cell Death and Proliferation by Flow Cytometry

CT-26 was arranged in 24-well plates and treated with free OXA and PLGA NPs (NPs 1, NPs 2, NPs 6, and NPs 7, 5 μg/mL and 25 μg/mL) for 24 and 48 h. For 3T3 cells, the same experimental design was performed during the 48 h of treatment. At each time point, the cells were labeled with Annexin V- fluorescein isothiocyanate (FITC) (BD Biosciences, San Jose, CA, USA) and 4,6-diamidino-2-phenylindole (DAPI) (Thermo Fisher Scientific, Cambridge, MA, USA), and analyzed with a flow cytometry.

For proliferation analyses, CT-26 and SW-480 cells were seeded in 12-well plates. The following day, the cells were treated with 25 μg/mL of free OXA and 5 μg/mL of PLGA NPs (NPs 1 and NPs 2) for 48 h. After treatment, the cells were incubated with the allophycocyanin (APC)-conjugated anti-mouse Ki-67 (1:100) (Thermo Fisher Scientific, Cambridge, MA, USA). The analyses were performed as described above.

### 2.6. Immunofluorescence, FADD, BCL-2, and Caspase-3 Activity

CT-26 cells and SW-480 cells were treated with OXA (5 μg/mL and 25 μg/mL) and PLGA NPs (NPs 1 and NPs 2, both 5 μg/mL) for 24 and 48 h. At each time point, CT-26 cells were incubated with the primary antibodies (Abcam, Burlingame, CA, USA), fas-associated protein with death domain (FADD) (ab24533), BCL-2 (ab32124), and caspase-3 (ab13847), and SW-480 cells were incubated with caspase-3 (ab13847). The primary antibody was detected with goat anti-rabbit Alexa Fluor 555 secondary antibody (ab150078; Abcam) and DAPI was used for nuclear staining. Specimens were examined with a Leica DM5500 B fluorescence microscope, equipped with a Leica DFC365 FX digital camera. Digital images were acquired and stored using Leica Application Suite X (LAS X) software.

### 2.7. Internalization of PLGA NPs by Cells and Visualization by Fluorescence Imaging

CT-26 cells were seeded on 12-mm coverslips placed at the bottom of a 12-well plate. After 24 h of incubation, NPs 1 and NPs 2 (5 µg/mL) were added. After 4 h and 24 h of incubation with NPs, the cells were stained with a membrane stain and the nuclei were counterstained with DAPI. For the visualization, a Leica DM5500 B fluorescence microscope was used as described above.

### 2.8. CRC Xenograft Models and Treatment Regimens

For xenographic tumor induction, a suspension of CT-26 cells (5 × 10^6^) was subcutaneously injected into the right flank of male Balb/c mice. The protocol was approved by the Committee on the Ethics of Animal Experiments of the UFRN (Universidade Federal do Rio Grande do Norte) (CEUA, permit number: 170.020/2019).

Once the tumor volume reached 3–4 mm [32], the animals were categorized into four groups (N = 8, each group) and treated intratumorally three times in 15 days with: (1) Control (CTRL) = 5 mg/kg saline solution, (2) OXA = 5 mg/kg, (3) NPs 1 = 5 mg/kg, and (4) NPs 2 = 5 mg/kg. Then, the tumor size was monitored every two days for 21 days or until the tumor reached a volume of 2000 mm^3^ [33,34]. Their size was calculated with the following equation [35]:

Volume = (length × width^2^ × 0.523)

Animals were euthanized with (80 mg/kg, i.p.) 2% thiopental (Cristália, São Paulo, Brazil) on day 21. Subcutaneous tumor masses were harvested and immediately frozen at −80 °C for qPCR analysis. Other tumors fragments were immersed in 10% paraformaldehyde for histopathological analysis.

### 2.9. Immunohistochemical Staining of FADD, APAF-1, and BCL-2

From tumors of each treatment group, 4-μm-thin tissue sections were cut using a microtome and transferred to gelatin-coated slides [36]. Tissue sections were incubated with primary antibodies anti-FADD (ab24533), anti-apoptotic protease activating factor 1 (APAF-1) (ab2001), and BCL-2 (ab32124; Abcam, Burlingame, CA, USA) at 4 °C overnight. Slices were washed with phosphate-buffered saline (PBS) and incubated with a streptavidin/Haptoglobin Related Protein (HRP)-conjugated secondary antibody (Biocare Medical, Concord, CA, USA). Immunoreactivity to the various proteins was visualized with a colorimetric-based detection kit following the protocol provided by the manufacturer (TrekAvidin-HRP Label + Kit from Biocare Medical, Pacheco, USA). Light microscopy (Nikon Eclipse 2000 equipped with Nikon DS-Fi2; Nikon Corporation, Tokyo, Japan) with a high-power objective (40×) was used to acquire digital images. The intensity of cell immunostaining was scored as follows: 1 = absence of positive cells, 2 = small number of positive cells or isolated cells, 3 = moderate number of positive cells, and 4 = large number of positive cells. Labelling intensity was evaluated by two previously trained examiners in a double-blind fashion.

### 2.10. Analysis of mRNA Expression

Total RNA was extracted from fragments of tumor tissue with a trizol reagent (Invitrogen Co., Carlsbad, CA, USA) and the SV Total RNA Isolation System (Promega, Madison, WI, USA). Real-time quantitative PCR analyses of *β*-actin, FADD, APAF-1, multidrug resistance protein 1 (MDR1), survivin, C-X-C chemokine receptor type 4 (CXCR4), and monocyte-derived chemokine (CCL22) mRNAs were performed with SYBR Green Mix in the Applied Biosystems1 7500 FAST system (Applied Biosystems, Foster City, CA, USA), according to a standard protocol with the primers listed in Table 2.

The standard PCR conditions were as follow: 50 °C for 2 min and 95 °C for 10 min, followed by 40 30-s cycles at 94 °C, a variable annealing primer temperature for 30 s, and 72 °C for 1 min. Mean threshold cycle (Ct) values were used to calculate the relative expression levels of the target genes for the experimental groups, relative to those in the negative control group; expression data were normalized relative to the housekeeping gene *β*-actin using the 2^−ΔΔCt^ formula.

### 2.11. Primary CRC Tissue Microarray

Biopsies were obtained from 180 patients undergoing surgical resection of CRC at the Cancer Referral Center of Natal, Brazil. Additional clinical information has been previously described together with the method to generate the tissue microarray (TMA) blocks [37]. This research was approved by the institutional committee (No. 030/0030/2006, 20th July, 2006).

For immunohistochemistry (IHC), anti-BCL-2, anti-caspase-8, and anti-Ki-67 antibody were used (Abcam, Burlingame, CA, USA) at a dilution of 1:1500. The number of positive cells within each TMA core was counted under a light microscope with a high-power magnification (40×).

BCL-2, caspase-8, and Ki-67 expression in the tumor tissue and surrounding stromal tissue was independently assessed by two researchers, who were blinded to the clinical data. Disease staging was performed according to the modified Dukes’ criteria classification [37].

### 2.12. Statistical Analysis

All in vitro experiments or in vivo were performed in triplicate and a one-way ANOVA was used, followed by Bonferroni’s post hoc test for parametric data and Kruskal–Wallis test followed by a Dunn’s multiple comparison test. A *p*-value of <0.05 was considered to be statistically significant (*p* < 0.05, *p* < 0.01, *p* < 0.001, and *p* < 0.0001). Statistical significance was measured using parametric testing of the TMA samples, assuming equal variance, with a standard *t*-test for two paired samples used to assess the difference between test and control samples, unless stated otherwise. The probability of survival over time compared to positivity of immunohistochemical labeling for Ki-67, caspase-8, and BCL-2 was estimated using Kaplan–Meier product limit survival curves with a log-rank (Mantel–Cox) comparison test.

## 3. Results

### 3.1. Preparation and Physicochemical Properties of PLGA NPs

**Figure 1:**
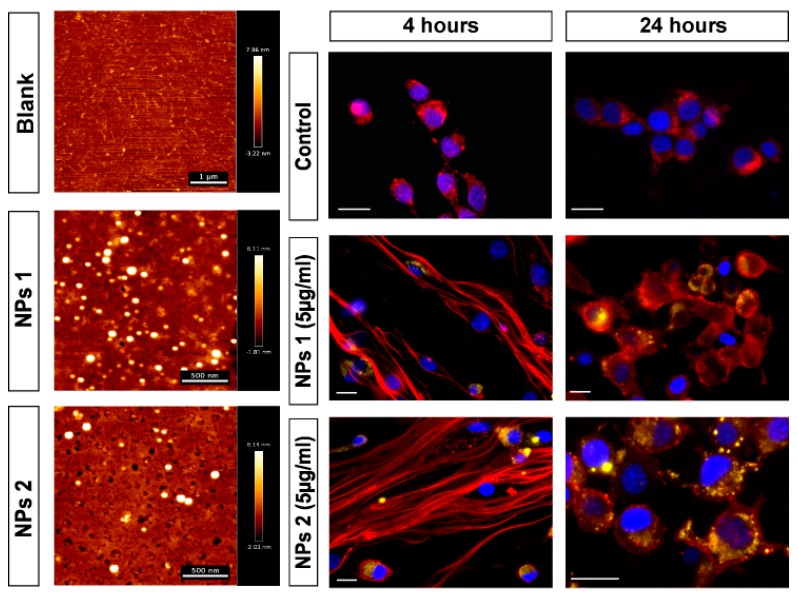
Representative AFM images of blank glass, NPs 1 and NPs 2 in alternating contact (AC) mode in air (scale bar = 500 µm) are showed in the left column of the panel. Fluorescence images in the right columns of the panel show the internalization of NPs by CT-26 cells. The nanoparticles (yellow) were detected in all treated groups and time points. Scale bar: 20 µm.

We loaded NPs with OXA and then studied their therapeutic potential (Table 1). Besides the empty NPs (control), each batch was a combination of the following compounds: OXA, RA, and CHO. CHO improved and facilitated the uptake of the PLGA NPs into cancer cells. First, the PLGA NPs were characterized to ascertain their size and surface charge (Table 1). The average size ranged from 400 nm to 800 nm in diameter. The average zeta potential ranged between −21.4 mV and −29.6 mV. Figure 1 shows two representative examples of AFM analysis of PLGA NPs, which revealed that all PLGA NPs were spherical in shape with a uniform size distribution.

### 3.2. Viability Assay

**Figure 2:**
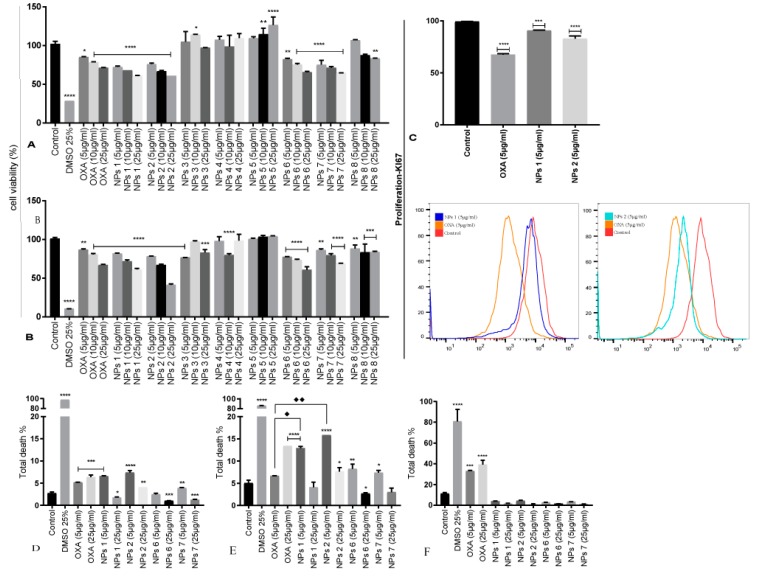
Cell viability (**A**,**B**), proliferation (**C**), total death for CT-26 for 24 h (**D**), total death for CT-26 for 48 h (E) and total death for 3T3 cells for 48 h (**F**). Mean cell proliferation of CT-26 cells treated with OXA and PLGA NPs for 24 h (**A**) and 48 h (**B**). Ki-67 immunostaining of CT-26 cells treated with OXA (5 μg/mL), NPs 1 (5 μg/mL), and NPs 2 (5 μg/mL) and compared to negative control for 48 h (**C**). The statistic of the treatments for the total death when compared to the negative control is displayed (* *p* < 0.05, ** *p* < 0.01, *** *p* < 0.001, and **** *p* < 0.0001). Comparison between OXA (5 μg/mL) and NPs 1 (5 μg/mL, ♦) as well as between OXA (5 μg/mL) and NPs 2 (5 μg/mL, ♦♦).

After incubation with the samples for 24 and 48 h, the viability of CT-26 was assessed by MTS assay in a concentration-dependent manner (Figure 2A,B). To assess the possible improvement of drug efficacy, two cell viability tests were performed: (1) With free OXA and (2) with CHO-coated PLGA NPs containing OXA and/or RA. The range of 5 μg/mL to 200 μg/mL of OXA highly reduced cell viability after 24 and 48 h (Figure S1). However, concentrations of 5, 10, and 25 μg/mL of this chemotherapeutic agent were closest to the half maximal inhibitory concentration (IC50). Therefore, these concentrations were used in a second step.

NPs 1, NPs 2, NPs 6, and NPs 7 reduced cell viability comparable to free OXA. Cell proliferation reduced in a dose-dependent manner, thereby, confirming the efficacy and successful uptake of our nanoparticulate systems (Figure 2A,B). The others system, i.e., NPs 3, NPs 4, and NPs 8, did not demonstrate satisfactory cytotoxicity for CT-26 cells because they did not have OXA in their composition. The formulation of NPs 5 (empty control) did not show cytotoxicity, confirming that PLGA is biocompatible and did not influence the results (Figure 2A,B).

**Figure 3:**
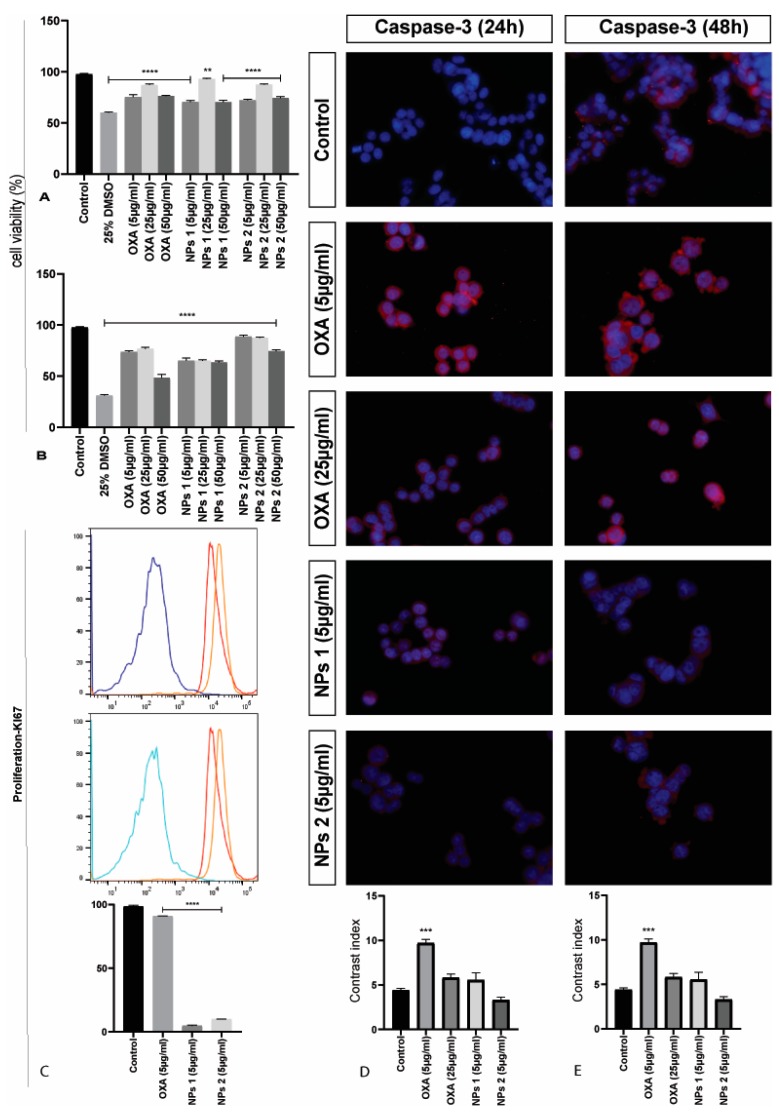
Cell viability, proliferation, and detection of caspase-3 of SW-480 cells. Mean cell proliferation of SW-480 cells treated with OXA and PLGA NPs for 24 h (**A**) and 48 h (**B**). All treatment groups were compared to the negative control group (**** *p* < 0.0001 and ** *p* < 0.01). Ki-67 immunostaining of SW-480 cells treated with OXA, NPs 1, and NPs 2, and compared to negative control for 48 h (**C**). All treatments were statistically significant (**** *p* < 0.0001). Representative photomicrographs of caspase-3 in SW-480 cells stained with 4,6-diamidino-2-phenylindole (DAPI) (blue) and anti-caspase-3 antibody (red). Contrast index for caspase-3 after 24 h (**D**) and 48 h (**E**) (*** *p* < 0.001). Scale bar: 50 µm.

To evaluate the effect of free OXA, NPs 1, and NPs 2 on the viability of the human CRC cell line SW-480, cells were treated with doses of 5, 25, and 50 μg/mL for 24 and 48 h. The results showed that free OXA as well as the NPs induced cell death, as determined by Hoechst labelling and flow cytometric analysis (Figure 3A,B). Thus, in conclusion, free OXA as well as NPs 1 and NPs 2 (5 μg/mL and 25 μg/mL) showed cytotoxicity in CT-26 and SW-480 cells after 24 and 48 h of incubation.

### 3.3. Detection of Apoptosis and Proliferation by Flow Cytometery

The dot plots generated by flow cytometric analysis show counts of cells with initial apoptosis (Annexin V-FITC-positive/DAPI-negative) in the lower right quadrant, while the upper right quadrant represents late apoptosis (Annexin V-FITC-positive/DAPI-positive) (Figures S2–S4). The total apoptosis was calculated with the sum of early (Q3) and late (Q2) apoptotic cells.

In CT-26 cells, the antitumor activity of OXA (5 μg/mL and 25 μg/mL) induced apoptosis after 24 h (*p* < 0.001, Figure 2D). However, after 48 h, only a concentration of 25 μg/mL OXA showed significant activity (*p* < 0.0001, Figure 2E). Similarly, NPs 1 (5 μg/mL, *p* < 0.001), NPs 2 (5 μg/mL and 25 μg/mL, *p* < 0.0001 and *p* < 0.01, respectively), and NPs 7 (5 μg/mL, *p* < 0.01) induced apoptosis after 24 h (Figure 2D). However, unlike free OXA, NPs 1 (5 μg/mL, *p* < 0.0001), NPs 2 (5 μg/mL and 25 μg/mL, *p* < 0.0001 and *p* < 0.05, respectively), NPs 6 (5 μg/mL, *p* < 0.01), and NPs 7 (5 μg/mL, *p* < 0.05) induced apoptosis after 48 h (Figure 2E). When compared to free OXA at the same concentration, NPs 1 (5 μg/mL, *p* < 0.0001) and NPs 2 (5 μg/mL, *p* < 0.0001) showed statistically significant antitumor activity after 48 h (Figure 2E). Importantly, our NPs did not induce apoptosis in nontumor 3T3 cells at any dose (Figure 2F). However, nontumor cells showed a significant death rate when exposed to free OXA (5 μg/mL and 25 μg/mL, *p* < 0.001 and *p* < 0.0001, respectively) after 48 h (Figure 2F).

A Ki-67 immunostaining was performed on CT-26 cells to evaluate the cell growth fraction after treatment with free OXA (5 μg/mL), NPs 1 (5 μg/mL), and NPs 2 (5 μg/mL), which was expressed in the G1, S, and G2/M cell cycle phases and was absent in resting (G0) cells. CT-26 cells treated with NPs 1 and NPs 2 showed a higher Ki-67 expression (*p* < 0.001 and *p* < 0.0001, respectively) than free OXA when compared to the negative control (*p* < 0.0001, Figure 2C). However, SW-480 cells treated with NPs 1 and NPs 2 exhibited a lower Ki-67 expression than free OXA when compared to the negative control (*p* < 0.0001, Figure 3C).

### 3.4. Immunofluorescence of FADD, BCL-2, and Caspase-3

To investigate the activated apoptosis pathway in CT-26 cells treated with free OXA and NPs 1 and NPs 2, three different proteins were investigated by means of immunofluorescence microscopy.

**Figure 4:**
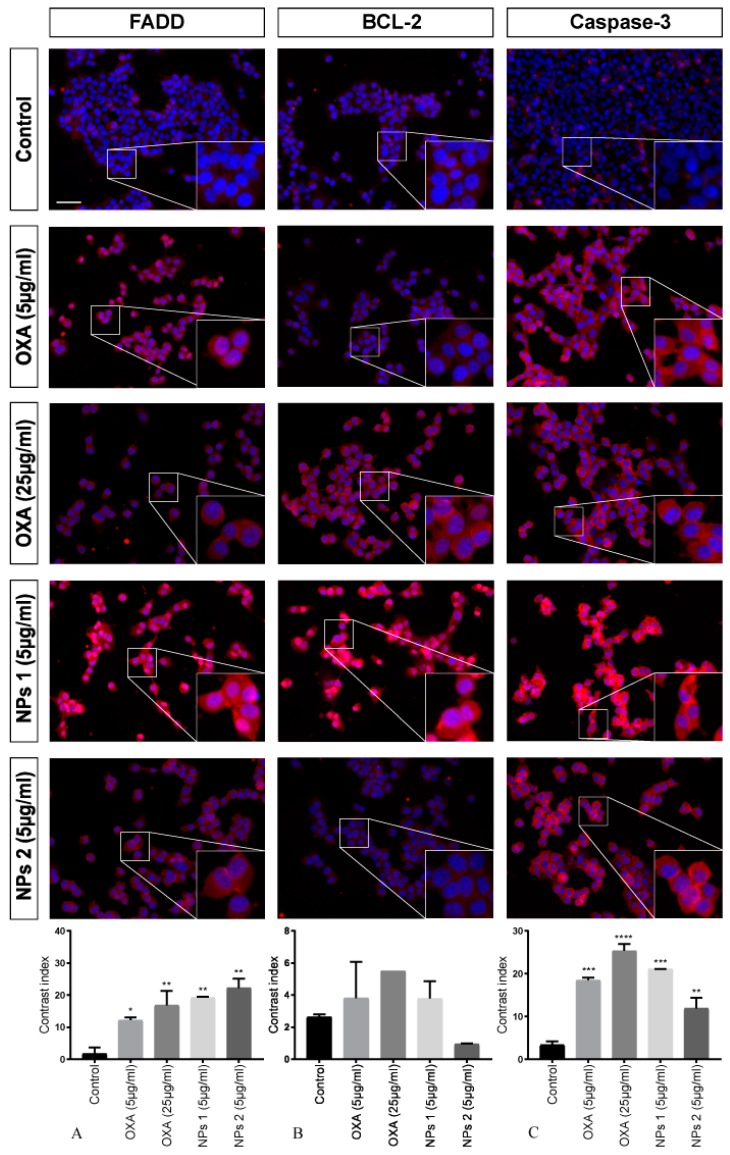
Detection of fas-associated protein with death domain (FADD), BCL-2, and caspase-3 after 24 h of treatment. CT-26 cells stained with DAPI (blue), anti-FADD, anti-BCL-2, and anti-caspase-3 antibodies (red). Contrast index for FADD, * *p* < 0.05, ** *p* < 0.01 (**A**); BCL-2, *p* > 0.05 (**B**); and caspase-3, ** *p* < 0.01, *** *p* < 0.001, **** *p* < 0.0001 (**C**). Scale bar: 50 µm.

After treating with OXA (5 μg/mL and 25 μg/mL), NPs 1 (5 μg/mL), and NPs 2 (5 μg/mL), antibody staining (FADD and caspase-3) was statistically significant when compared to the control for all samples after 24 h. However, BCL-2 staining did not show significant results (*p* > 0.05, Figure 4). For FADD, it was *p* < 0.05, *p* < 0.01, *p* < 0.01, and *p* < 0.01, respectively, and for caspase-3 it was *p* < 0.001, *p* < 0.0001, *p* < 0.001, and *p* < 0.01, respectively.

**Figure 5:**
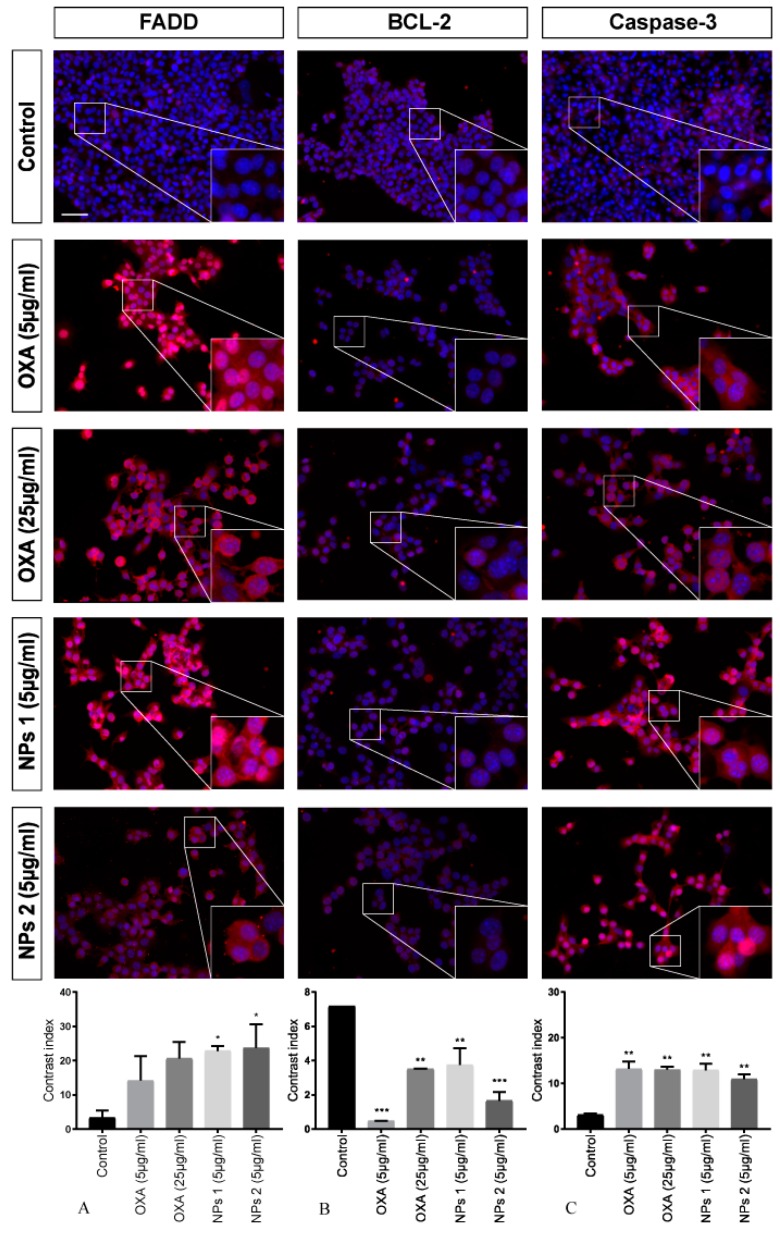
Detection of FADD, BCL-2, and caspase-3 after 48 h of treatment. CT-26 cells stained with DAPI (blue), anti-FADD, anti-BCL-2, and anti-caspase-3 antibodies (red). Contrast index for FADD, * *p* < 0.05 (**A**); BCL-2, ** *p* < 0.01, *** *p* < 0.001 (**B**); and caspase-3, ** *p* < 0.01 (**C**). Scale bar: 50 µm.

Our analysis showed significant FADD staining for NPs 1 (5 μg/mL, *p* < 0.05) and NPs 2 (5 μg/mL, *p* < 0.05) only 48 h after treatment. However, a decrease in BCL-2 immunoreactivity for all treatments (*p* < 0.001, *p* < 0.01, *p* < 0.01, *p* < 0.001) was observed. When compared to the control, caspase-3 staining of CT-26 treated with OXA, NPs 1, and NPs 2 was significant (*p* < 0.01 for all treatments, Figure 5).

When caspase-3 staining was evaluated in SW-480 cells, a higher expression of caspase-3 was perceived in cells treated with OXA (5 μg/mL, *p* < 0.01) when compared to control cells after 24 and 48 h (Figure 3D,E). However, OXA (25 μg/mL), NPs 1 (5 μg/mL), and NPs 2 (5 μg/mL) did not show significant caspase-3 expression when compared to the control.

### 3.5. Internalization of NPs by CT-26 Cells

Internalization of NPs 1 and NPs 2 by CT-26 cells was studied using fluorescence microscopy (Figure 1). After 4 h and 24 h of incubation, the cell membranes were stained to visualize internalization of NPs 1 and NPs 2 by cells. NPs 1 and NPs 2 showed accumulation within the cells at both time points.

### 3.6. In Vivo Study

**Figure 6:**
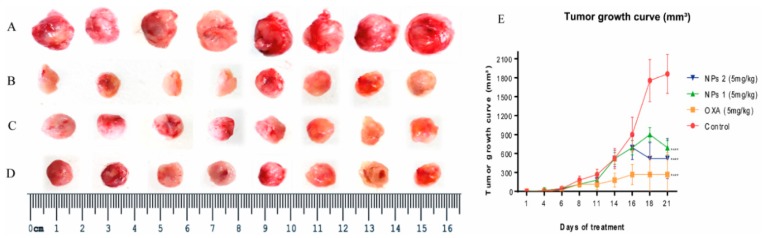
Morphology of all the tumors collected from (**A**) control, (**B**) OXA (5 mg/kg), (**C**) NPs 1 (5 mg/kg), and (**D**) NPs 2 (5 mg/kg) mice. Tumor growth curve of the Balb/c xenografts with different treatments (**E**). All treatment groups were compared to the negative control group (**** *p* < 0.0001).

**Figure 7:**
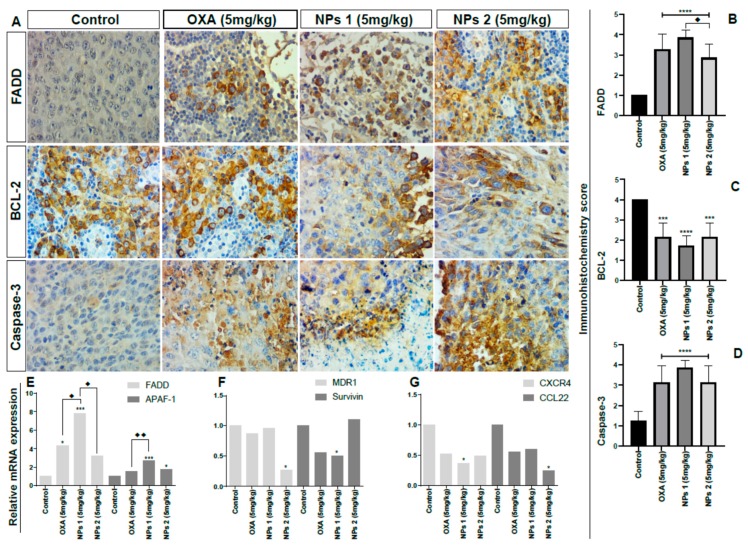
Analysis of apoptosis, drug resistance, and metastasis factors. Representative photomicrographs of immunohistochemistry of tumor fragments of mice receiving different treatments (**A**). Immunohistochemistry score by anti-FADD (**B**), anti-BCL-2 (**C**), anti-caspase-3 (**D**), relative messenger ribonucleic acids (mRNA) expression by RT-PCR for FADD and apoptotic protease activating factor 1 (APAF -1) (**E**), multidrug resistance protein 1 (MDR1) and survivin (**F**), and C-X-C chemokine receptor type 4 (CXCR4), and monocyte-derived chemokine (CCL22) (**G**). All treatment groups were compared to the negative control group (**** *p* < 0.0001). Comparison between OXA (5 mg/kg) and NPs 1 (5 mg/kg, ♦ *p* < 0.05) as well as between OXA (5 mg/kg) and NPs 2 (5 mg/kg, ♦♦ *p* < 0.01) was also performed (*p* < 0.0001 for both). Magnification: 40×.

**Figure 8:**
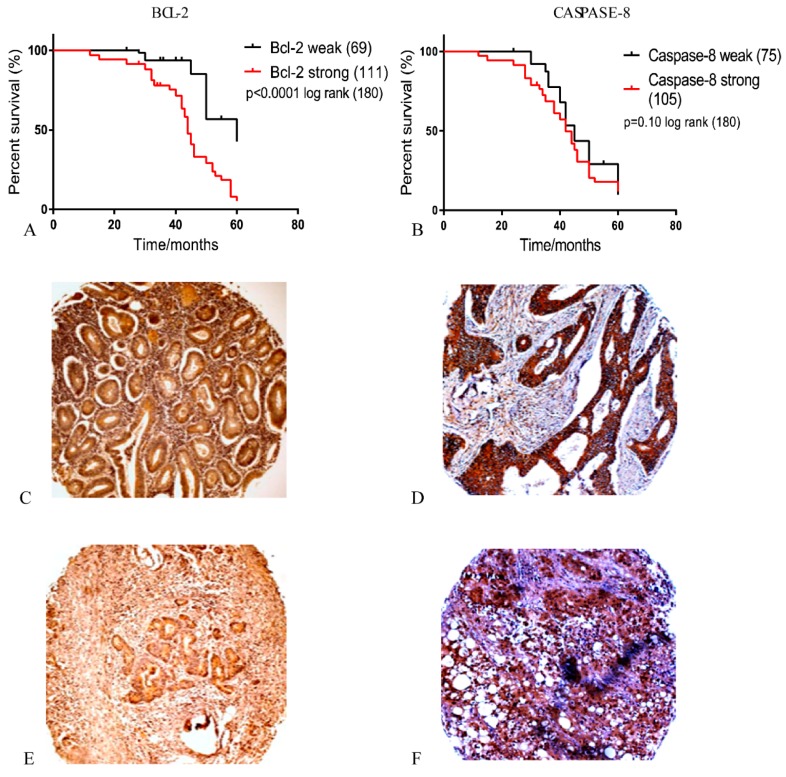
Expression of BCL-2 and caspase-8 in primary colorectal tumors. (**A**) Kaplan–Meier survival curve estimated by BCL-2 staining in colorectal cancer (CRC) tumors (log-rank × 2 = 30.75, *p* < 0.0001), (**B**) Kaplan–Meier survival estimated by caspase-8 staining in CRC tumors (log-rank × 2 = 2.61, *p* = 0.10). Immunohistochemical staining for BCL-2 and caspase-8 in colorectal carcinoma. Colorectal adenocarcinoma with a high modified Dukes’ classification (“3” and “4”) showing: (**C**) Strong cytoplasmic staining of BCL-2, and (**E**) weak BCL-2 cytoplasmic staining. Strong cytoplasmic staining of caspase-8 (**D**), and (**F**) weak caspase-8. Magnification: 40×.

To confirm whether NPs 1 and NPs 2 were more efficient DDSs to downregulate apoptosis pathways, drug resistance, and metastasis factors in vivo when compared to free OXA, the expression of FADD, APAF-1, BCL-2, caspase-3, MDR1, survivin, CXCR4, and CCL22 was observed in tumors of Balb/c mice using qPCR and/or immunohistochemistry. As shown in Figure 6, tumor growth and volume decreased in groups treated with free OXA, NPs 1, and NPs 2 (5 mg/kg, *p* < 0.0001). After 15 days of treatment with free OXA (5 mg/kg), NPs 1 (5 mg/kg), and NPs 2 (5 mg/kg), immunohistochemical analyses revealed an increased expression of FADD (Figure 7A,B, *p* < 0.0001) and caspase-3 (Figure 7A,D, *p* < 0.0001), and a decreased expression of BCL-2 (Figure 7A and Figure 8C, *p* < 0.0001 for NPs 1 and *p* < 0.001 for OXA and NPs 2) in tumors of Balb/c mice when compared to the control group. A comparison between the different treatment groups showed a higher expression of FADD and caspase-3 in tumors of groups treated with NPs 1 (Figure 7B and Figure 8D) than in groups treated with free OXA and NPs 2. On the other hand, BCL-2 had a lower expression in groups treated with NPs 1 (Figure 7C) than in the other two groups. Indeed, genes of FADD and APAF-1 were evaluated in tumors by means of RT-PCR after 15 days of treatment. From the gene expression analysis, it was observed that the increase of FADD was statistically significant in groups treated with free OXA (5 mg/kg) and NPs 1 (5 mg/kg) when compared with the control (Figure 7E, *p* < 0.05 and *p* < 0.001, respectively). The increase of APAF-1 was statistically significant in groups treated with NPs 1 (5 mg/kg) and NPs 2 (5 mg/kg) when compared to the control (Figure 7E, *p* < 0.001 and *p* < 0.05, respectively). When the gene expression of FADD and APAF-1 is compared between the treatment groups, it is apparent that their expression was higher after treatment with NPs 1 than after treatment with either free OXA or NPs 2 (Figure 7E).

The gene expression analysis of drug resistance and metastasis factors was also evaluated by RT-PCR. Based on Figure 7F, the expression of MDR1 and survivin genes, which are involved in drug resistance, was reduced after treatment with NPs. The MDR1 gene expression decreased after treatment with NPs 2 (5 mg/kg, *p* < 0.05), while the survivin gene expression decreased after treatment with NPs 1 (5mg/kg, *p* < 0.05). As metastasis is one of the biggest concerns of cancer management strategies, we investigated two genes from chemokines related to metastasis by means of RT-PCR. When compared to the control group, NPs 1 was able to reduce CXCR4 gene expression while NPs 2 decreased the CCL22 gene expression (Figure 7G, both *p* < 0.05).

### 3.7. Expression of Anti-Apoptosis and Pro-Apoptosis Proteins in Primary Colorectal Tumors

Apoptosis is associated with several other processes of tumor progression, like drug resistance, cell proliferation, and metastasis. Here, we evaluated if the expression of BCL-2, caspase-8, and Ki-67 would be associated with poor prognosis in tumors with low (1/2) and high (3/4) modified Dukes’ classification of the patients in primary CRC. High expression of BCL-2 was an indication of recurrence of the CRC (*p* = 0.0001, Figure 8). In addition, patients who showed high expression of caspase-8 indicated a greater tendency towards death within 60 months of follow-up, although our data were not statically significant (*p* = 0.10, Figure 8). Furthermore, high expression of BCL-2 and caspase-8 was associated with more aggressive tumors, as represented by lymph node involvement (*p* = 0.0002 for both), modified Dukes’ criteria grade (*p* = 0.0001 and *p* = 0.07, respectively), and proliferation by Ki-67 (*p* = 0.04 and *p* = 0.0001, respectively, Table 3). These results show that the synthesis of new drug systems to specifically target apoptosis, especially in human primary tumor of aggressive cancers, is necessary as part of a novel cancer management strategy.

## 4. Discussion

Evasion of apoptosis is one of the major causes of tumor progression. In this study, we identified increased expression of BCL-2 and caspase-8 in human CRC. In addition, we demonstrated that increased expression of BCL-2 and caspase-8 is associated with proliferation (Ki-67), a high invasion grade, and a positive lymph node status. This suggests a growth advantage of tumor cells with high expression of caspase-8 in the pathogenesis of CRC, which is consistent with previous studies [38,39,40,41]. However, previous studies showed that inactivating mutations of the caspase-8 genes are rare in human colorectal carcinomas [42]. There is growing evidence that caspase-8 does not just act as an inducer of apoptosis, but also in metastasis [43]. Induction of cell death is one of the challenges for the development of new drugs, especially in aggressive tumors with a high level of mortality.

Based on evidences that tumor progression is associated with failing cell control mechanisms, we designed OXA-loaded PLGA NPs combined with RA and CHO to assess the improvement of the antitumor activity of OXA. Firstly, we evaluated the cytotoxicity of OXA-containing NPs in CT-26 cells. The data showed that OXA-containing NPs 1, NPs 2, NPs 6, and NPs 7 possess a cytotoxic activity (after 24 and 48 h), which was similar to the free drug. However, NPs 1 and NPs 2 exhibited a higher cytotoxic activity than others NPs after 48 h. According to these results, we decided to assess the cytotoxicity of NPs 1 and NPs 2 in SW-480 cells. Both NPs showed to have cytotoxic activity in this cell line. These results revealed that RA and CHO helped OXA to inhibit the cell viability in CT-26 as well as SW-480 cells, probably by increasing the availability or potentializing the cytotoxic effects of OXA inside of cells [23,44,45].

OXA is a third-generation platinum drug, which inhibits DNA replication [46]. However, the obtained efficacy is suboptimal due to the aggressive side effects OXA and drug resistance of cancer cells [46,47,48]. The pro-apoptotic activities of NPs and OXA were analyzed by flow cytometry for both CT-26 and nontumor cells. NPs 1, NPs 2, NPs 6, and NPs 7 had pro-apoptotic activity at a concentration of 5 μg/mL in the tumor cell line at 24 and 48 h. However, NPs 1 and NPs 2 exhibited a higher pro-apoptotic activity than higher concentrations of free OXA. Interestingly, they also reduced the total apoptosis in the nontumor cell line. Previous studies reported that NPs-encapsulated chemotherapeutic agents induce apoptosis in several tumor cell lines without interfering with nontumor cell lines [49,50]. This result indicates that it is possible to use lower concentrations of OXA when it is encapsulated in a nanoparticulate DDS. In addition, it is possible to combine OXA with CHO and RA. Due to their high proliferation rate, tumor cells require higher amounts of CHO than nontumor cells. Thus, coating with CHO facilitates the internalization of NPs and, thereby, increases the delivery of OXA into tumor cells. This improves the efficiency of the system and decreases the adverse effects that are related to the cumulative impact of OXA on patients [12,23,51]. Our study contributed to this knowledge and, in addition, we did not observe significant cell death in nontumor cells. This indicates that these nanoparticulate DDSs (NPs 1 and NPs 2) can have a protective effect on nontumor cells, while selectively targeting tumor cells.

High uptake of NPs by tumor cells and effective intracellular drug release are necessary to achieve an enhanced therapeutic effect in clinic trials. The uptake of NPs 1 and NPs 2 by CT-26 cells was investigated after 4 h and 24 h, which revealed that our NPs were mainly located in the cytoplasm close to the nucleus. It is worth mentioning that the CHO-coated NPs combined with RA (NPs 1 and NPs 2) showed rapid uptake and improved therapeutic efficacy through induction of apoptosis in CRC cells [23,52]. Therefore, our results indicate the importance of CHO for the increased capture and internalization of DDS in the treatment of CRC [23,53].

The pro-apoptotic activity of free OXA (5 μg/mL and 25 μg/mL), NPs 1 (5 μg/mL), and NPs 2 (5 μg/mL) in CT-26 cells was confirmed by immunoreactivity of caspase-3, FADD, and BCL-2, which are involved in the extrinsic and intrinsic apoptosis pathway. The extrinsic pathway was activated by all compounds within 24 h, but only NPs 1 and NPs 2 induced a positive immunoreactivity to FADD after 48 h. These results show that our OXA-containing nanoparticulated DDS exhibits a prolonged activity as compared to the free drug, which suggests an increased bioavailability inside the cancer cells [45,51,52]. Since immunoreactivity for BCL-2 was observed only after 48 h, our data suggests that the NPs act primarily by activating the extrinsic pathway. Previous studies give evidence for a strong correlation between intrinsic apoptosis and cell proliferation in tumor progression [44,54]. The expression of Ki-67 is strongly associated with (tumor) cell proliferation and growth, and is widely used in routine pathological investigations as a proliferation marker [55]. Owing to high cell proliferation, frequently associated with the Ki-67 protein labeling index, Ki-67 may be a promising factor for targeted molecular therapies [55]. In this study, NPs 1 (5 μg/mL) and NPs 2 (5 μg/mL) strongly decreased the expression of Ki-67 in SW-480 cells but not in CT-26 cells. Interestingly, this result corroborates findings in the literature by describing the anti-apoptotic protein BCL-2 as an inhibitor of p53, a pro-apoptotic and suppressor protein [56]. Since mutated p53 can also induce a G2/M cell cycle and stimulate Ki-67, our NPs 1 and NPs 2 were able to induce extrinsic apoptosis, which has an independent path of BCL-2 and p53 and showed that the BCL-2-p53-Ki-67 path is common in aggressive tumors [57]. In our study, we found that NPs 1 and NPs 2 induced apoptosis via the extrinsic path and blocked the proliferation of cancer cells, thereby, suggesting that the NPs act on different signaling pathways.

With the aim to observe the efficiency of NPs in an animal model, we inoculated CT-26 cells subcutaneously in the flank of Balb/c mice and, when the tumors reached 4 mm, the mice were treated with free OXA, NPs 1, and NPs 2 (all 5 mg/kg), respectively, 3 times over a period of 2 weeks. Based on the expression of FADD, BCL-2, and caspase-3, our in vivo results suggested that NPs 1 induced apoptosis more than NPs 2, especially through the extrinsic pathway, corroborating with our in vitro results. One of the advantages of RA combined with OXA and CHO, is that CHO increases internalization of nanoparticles as described above and, therefore, increased amounts of RA can act directly on the regulation of apoptotic pathway proteins and at the tumor microenvironment as demonstrated by Watabe et al. [53].

High levels of CXCR4, a receptor correlated with tumor malignancy, and CCL22, a member of the chemokine family, are related to migration, invasion, and metastasis in various tumors, leading to a poor prognosis and malignant progression [58,59,60,61]. Our results show that NPs 1 and NPs 2 decrease CXCR4 and CCL22 expression in the primary microenvironment, which indicates that our nanoparticulated DDSs act as important modulators of metastatic sites. Drug resistance to OXA is a problem, which reduces its effectiveness and increases the chances of metastasis, thereby, limiting tumor treatment with this drug. In this study, when analyzing primary tumors treated with free OXA as well as NPs 1 and NPs 2, we observed downregulation of multidrug resistance (MDR), such as, MDR1 expression by NPs 2 and survivin by NPs 1. This proves that encapsulation of drugs decreases drug resistance since NPs were taken up by endocytosis by passing drug efflux pumps which are altered in tumor cells [62,63]. Drug efflux pumps expressed on human cancer cells majorly contribute to MDR, especially those related to P-gp also known as multidrug resistance protein 1 (MDR1) and surviving. [64] These results suggest that CHO functionalized PLGA nanoparticles loaded with anti-cancer drug OXA and chemosensitizer RA enhanced therapeutic potential by modulating MDR of tumor cells through RA and enhanced the anticancer activity of DDSs. Dual drug loaded nanoparticles revealed better therapeutic efficacy with enhanced expression or downregulation of pro-apoptotic/anti-apoptotic proteins and downregulation of metastastic factors, such as CXCR4 and CCL22.

An upregulation of apoptosis in the microenvironment of human primary tumors is the key point to reduce proliferation and metastasis and, consequently, improve the survival of patients. Here, we designed a system of OXA-loaded NPs combined with RA and CHO to study their efficiency in inducing apoptosis and regulating proliferation, drug resistance, and metastatic factors. Taken together, the data presented in this study indicated that nanoparticulate DDSs (NPs 1 and NPs 2) possess antitumor activity in CRC cells in vitro and in vivo, which is preferentially mediated by the extrinsic apoptosis pathway. In vivo results also suggest that NPs 1 and NPs 2 downregulate resistance to OXA and metastasis factors. Cytoprotective activity based on the non-induction of cell death in nontumor cells is combined with the antitumor activity and modulation of the tumoral microenvironment, indicating that these systems are safe candidates for drug delivery that can be used for the treatment of cancer with decreased adverse effects.

## Figures and Tables

**Table 1:** Physicochemical properties of Poly(D,L-Lactide-co-Glycolic Acid) (PLGA) nanoparticles (NPs). Determination of retinoic acid (RA) and oxaliplatin (OXA) content, size distribution, and zeta-potential of PLGA NPs. Particles were characterized by Dynamic Light Scattering (DLS) and zeta-potential measurements. Particle size data represent the mean value ± standard deviation (SD) of dynamic light scattering data. Zeta-potential data represent the mean value ± SD of 10 readings. OXA and RA contents of PLGA NPs were determined by particle digestion and measured by reversed-phase high-performance liquid chromatography (RP-HPLC) analysis.

Samples	Loading Oxaliplatin Efficiency (µg/mg NPs)	Loading Retinoic Acid Efficiency (µg/mg NPs)	Size ± SD (nm)	PDI (Polydispersity Index)	Zeta Potential ± SD (mV)
NPs 1 (OXA, RA)-CHO	44	40	801.7 ± 165.4	0.598	−21.4 ± 8.4
NPs 2 (OXA)-CHO	48	--	678.3 ± 118.5	0.694	−25.8 ± 15.9
NPs 3 (RA)-CHO		46	539.8 ± 87.6	0.438	−28.5 ± 16.1
NPs 4 (empty)-CHO	--	--	443.1 ± 27.1	0.253	−23.6 ± 9.13
NPs 5 (empty)	--	--	496.7 ± 35.35	0.255	−28.8 ± 8.6
NPs 6 (OXA)	55	--	391.5 ± 60.53	0.182	−29.6 ± 9.9
NPs 7 (OXA, RA)	46	44	505.6 ± 64.30	0.199	−27.6 ± 42.1
NPs 8 (RA)	--	50	514.8 ± 106.7	0.136	−25.7 ± 10.8

**Table 2:** Primer sequences used for PCR.

mRNA	Oligonucleotides Primers		Temperature
*β*-actin	5′-AAC-TTT-GGC-ATCGTG-GAA-GG-3′	5′-GTGGATGCAGGGATGATGTTC-3′	60 °C
FADD	5′-AGAAGAAGAACGCCTCGGTG-3′	5′-GCTCACAGATTCCTGGGCTT-3′	56.5 °C
APAF-1	5′-TTCCAGTGGCAAGGACACAG-3′	5′-CCACTCTCCACAGGGACAAC-3′	56.8 °C
MDR1	5′-TCAGCAACAGCAGTCTGGAG-3′	5′-ACTATGAGCACACCAGCACC-3′	55.2 °C
Survivin	5′-AGAACAAAATTGCAAAGGAGACA-3′	5′-GGCATGTCACTCAGGTCCAA-3′	55.2 °C
CXCR4	5′-ACCTCGGTGTCCTCTTGCTGTCCA-3′	5′-GCTTGACGTTGGCTCTGGCGATGT-3′	56.5 °C
CCL22	5′-GAGACAACAGTGGTCCCAGG-3′	5′-CTGGCACTGTCAATCCCTGT-3′	56.8 °C

**Table 3:** Clinicopathological characteristics of CRC patients in relation to caspase-8 and BCL-2 expression. Distributions of tumor caspase-8 and BCL-2 expression categorizations according to clinical-pathological and Ki-67 expression (percentages in parenthesis). High expression of caspase-8 was associated with higher tumor grade, lymph node metastasis, and proliferation (Ki-67), while high expression of BCL-2 was associated with higher tumor grade and lymph node metastasis. Fisher’s exact = 1, chi-square = 2.

Clinicopathological Characteristics of CRC Patients	Caspase-8 (*n* = 180)			BCL-2 (*n* = 180)		
	Weak	Strong	*p* Value	Weak	Strong	*p* Value
**Number of Patients**	75 (41.6%)	105 (58.3%)	-	69 (38.33%)	111 (41.67%)	-
**Lymph Node Status**			0.0002 ^1^			0.0002 ^1^
**Negative**	15(20%)	03(2.86%)		39(56.52%)	23(20.72%)	
**Positive**	60(80%)	102(97.14%)		30(43.48%)	88(79.28%)	
**Modified Dukes’ Criteria Grade**			0.0001 ^1^			0.07 ^1^
**Low (I and II)**	25(33.3%)	07(6.67%)		41(59.42%)	35(31.53%)	
**High (III and IV)**	50(66.7%)	98(93.33%)		28(40.58%)	76(68.47%)	
**Ki-67**			0.04 ^2^			0.0001 ^2^
**<10%**	02(2.7%)	02(1.9%)		09(13.05%)	24(21.62%)	
**11–25%**	18(24%)	11(10.48%)		11(15.94%)	52(46.85%)	
**>25%**	55(73.3%)	92(87.62%)		49(71.01%)	35(31.53%)	

## Data Availability

The raw/processed data required to reproduce these findings cannot be shared at this time as the data also forms part of an ongoing study.

## Supplementary Materials

The following are available online at https://www.mdpi.com/1999-4923/12/2/193/s1, Figure S1. Mean cell proliferation of CT-26 cells treated with OXA for 24 h. (A) and 48 (B) hours. The concentrations used were: 5 μg/mL, 10 μg/mL, 25 μg/mL, 50 μg/mL, 100 μg/mL, and 200 μg/mL. All treatment groups were compared to the negative control group (**** p < 0.0001), Figure S2. Flow cytometry to determine apoptosis. Dot plots of flow cytometry with the effect of different doses of OXA, DMSO, and NPs on early and late apoptotic CT-26 cells after 24 h. are displayed, Figure S3. Flow cytometry to determine apoptosis. Dot plots of flow cytometry with the effect of different doses of OXA, DMSO, and NPs on early and late apoptotic CT-26 cells at 48 h. are displayed, Figure S4. Flow cytometry to determine apoptosis. Dot plots of flow cytometry with the effect of different doses of oxaliplatin, DMSO, and NPs, on early and late apoptosis in 3T3 cells at 48 h. are displayed.
