# Supplementary material for: Effect of Oxaliplatin-Loaded Poly (d,l-Lactide-co-Glycolic Acid) (PLGA) Nanoparticles Combined with Retinoic Acid and Cholesterol on Apoptosis, Drug Resistance, and Metastasis Factors of Colorectal Cancer
Source: Pharmaceutics. 2020 Feb 23;12(2):193. doi: 10.3390/pharmaceutics12020193 (PMC7076533; doi:10.3390/pharmaceutics12020193)
Supplement: Supplementary file 1 [file pharmaceutics-12-00193-s001.pdf]

pharmaceutics

MDPI

Supplementary Materials: Effect of Oxaliplatin-Loaded Poly (D,L-Lactide-*co*-Glycolic Acid) (PLGA) Nanoparticles Combined with Retinoic Acid and Cholesterol on Apoptosis, Drug Resistance, and Metastasis Factors of Colorectal Cancer

Ana Luiza C. de S. L. Oliveira [1,2], Raimundo Fernandes de Araújo Júnior [1,2,3,*], Thaís Gomes de Carvalho [1,2], Alan B. Chan [4], Timo Schomann [2,4], Filippo Tamburini [4], Lioe-Fee de Geus-Oei [5] and Luis J. Cruz [2,*]

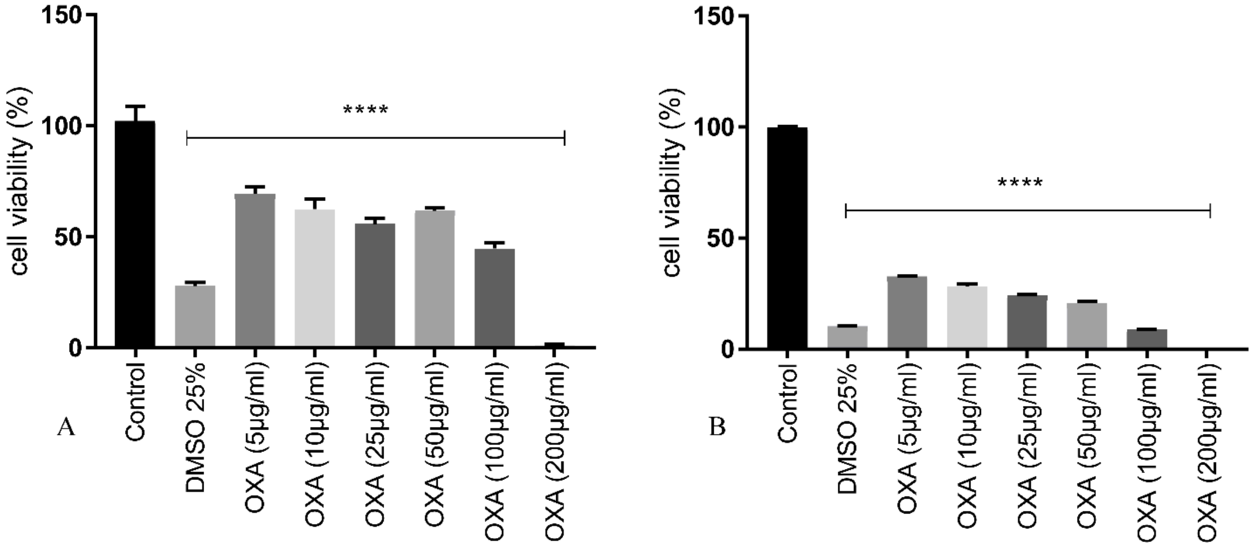

Figure S1. Mean cell proliferation of CT-26 cells treated with OXA for 24 hours (**A**) and 48 (**B**) hours. The concentrations used were: 5 µg/mL, 10 µg/mL, 25 µg/mL, 50 µg/mL, 100 µg/mL, and 200 µg/mL. All treatment groups were compared to the negative control group (****p < 0.0001).

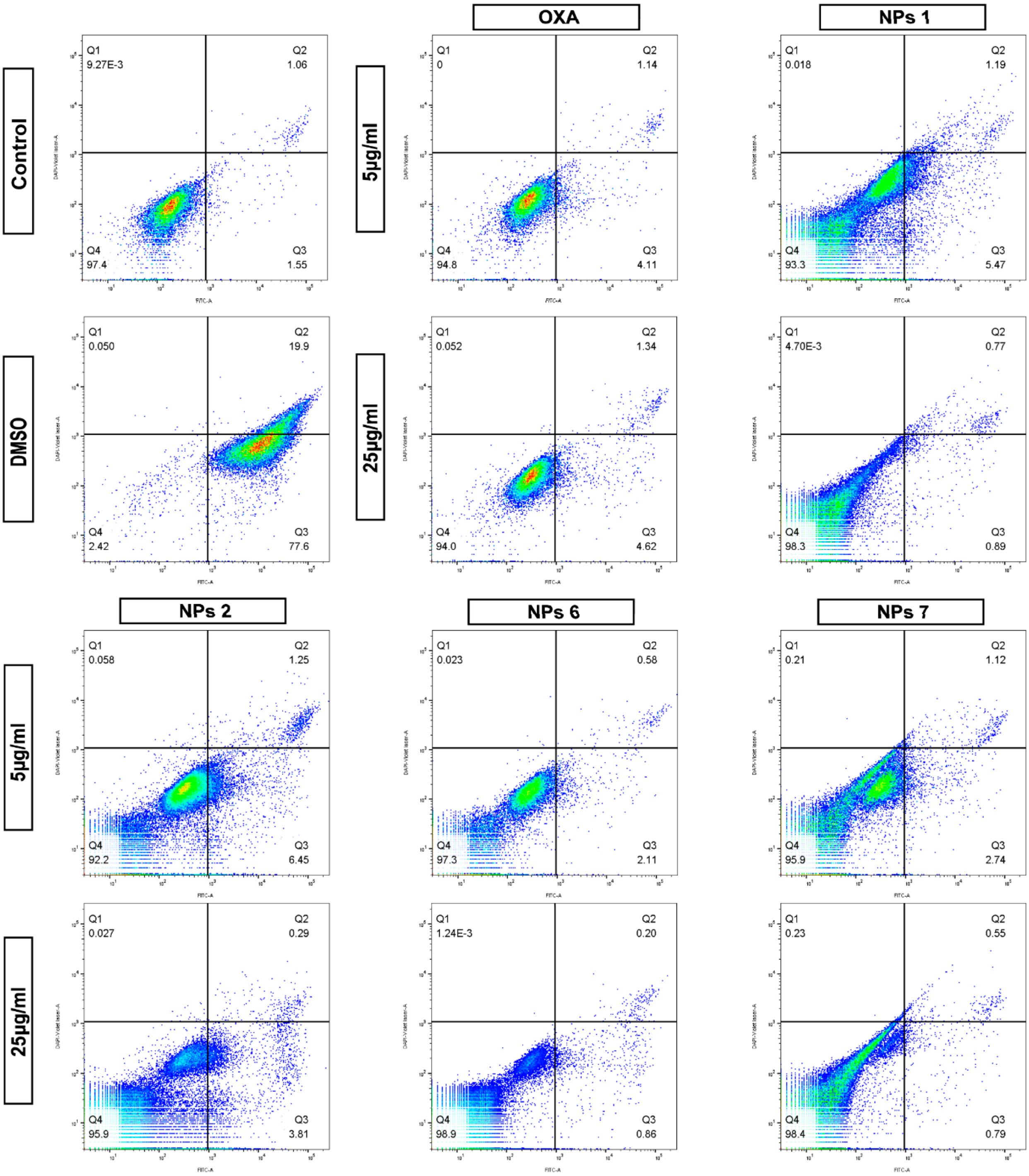

Figure S2. Flow cytometry to determine apoptosis. Dot plots of flow cytometry with the effect of different doses of OXA, DMSO, and NPs on early and late apoptotic CT-26 cells after 24 hours are displayed.

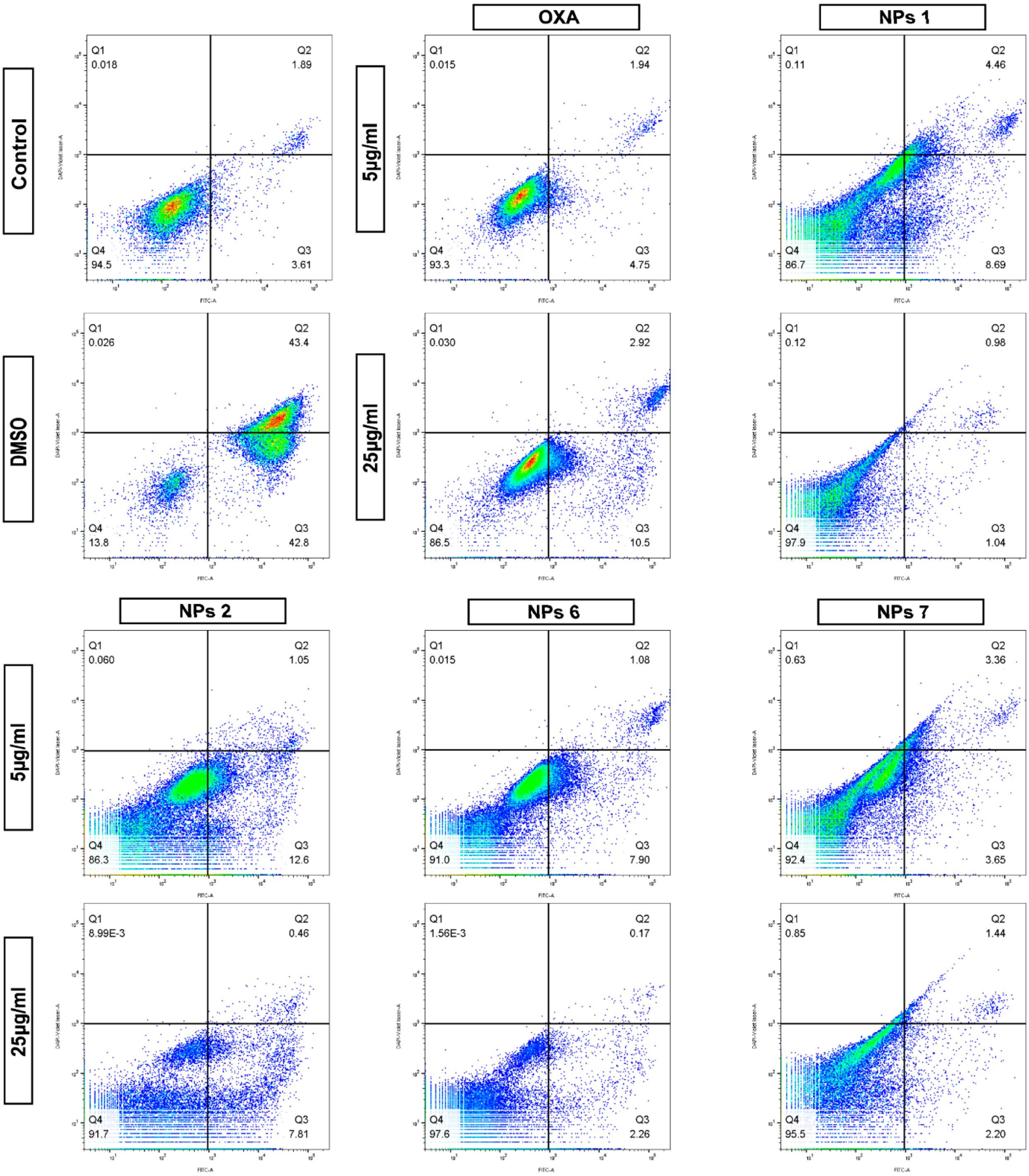

Figure S3. Flow cytometry to determine apoptosis. Dot plots of flow cytometry with the effect of different doses of OXA, DMSO, and NPs on early and late apoptotic CT-26 cells at 48 hours are displayed.

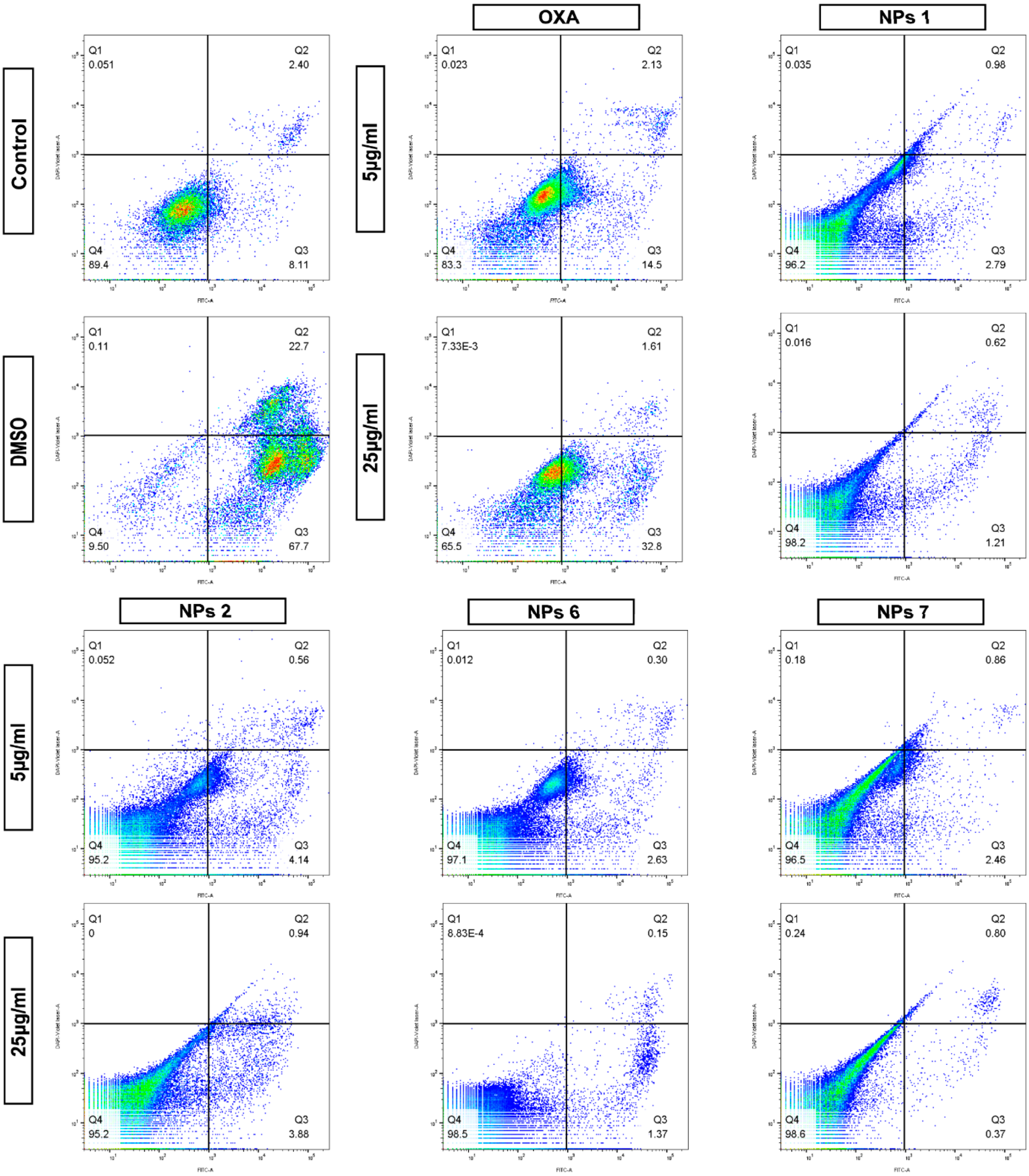

Figure S4. Flow cytometry to determine apoptosis. Dot plots of flow cytometry with the effect of different doses of oxaliplatin, DMSO, and NPs, on early and late apoptosis in 3T3 cells at 48 hours are displayed.
